# Radium Treatment of Cancer

**Published:** 1915-05

**Authors:** 


**Radium Treatment of Cancer.** Rupp, "Deutsche Medical Woch.," December 17, 1914, cites a personal case of large (inoperable?) genital cancer cured by radium, but also quotes Bumm as having first rayed operable cancers and then extirpated them and found cancer cells and nests in an active state, although within the zone of permeation by rays, 4 to 5 c. m. Hence he believes that the general policy should be to operate first and treat with emanations afterward.

